# PiCCO hemodynamic parameters in cardiogenic shock: prediction of LVEF, NT-proBNP and MACE based on XGBoost machine learning model

**DOI:** 10.3389/fmed.2025.1683425

**Published:** 2025-10-15

**Authors:** Jieyun You, Tianwen Wei, Yue Yu, Jing Huang, Yuxiao Sun, Wei Guo, Qi Zhang

**Affiliations:** Department of Cardiology, Shanghai East Hospital, School of Medicine, Tongji University, Shanghai, China

**Keywords:** XGBoost machine learning model, pulse index continuous cardiac output, left ventricular ejection fraction, N-terminal pro-brain natriuretic peptide, major adverse cardiovascular events

## Abstract

**Introduction:**

This study used the Extreme Gradient Boosting (XGBoost) machine learning model to conduct an in-depth analysis of the potential relationship between pulse index continuous cardiac output (PiCCO) and multiple clinical prognostic indicators, including left ventricular ejection fraction (LVEF), N-terminal pro-brain natriuretic peptide (NT-proBNP) levels, and 30-day major adverse cardiovascular events (MACE), in patients with cardiogenic shock. The aim of this study was to investigate the predictive ability of PiCCO hemodynamic parameters and the relative contribution features based on the XGBoost model.

**Methods:**

Multi-class receiver operating characteristic (ROC) curves explored that the XGBoost prediction model performed extremely well about LVEF and NT-proBNP. Further SHapley Additive explanation (SHAP) value analysis revealed the contributions of different PiCCO hemodynamic parameters.

**Results:**

Features such as CI (cardiac index), CPI (cardiac power index), and SVRI (systemic vascular resistance index) showed significant positive effects on the prediction of LVEF and NT-proBNP. In terms of MACE, dPmax (index of the left ventricular contractility), CFI (cardiac function index), and GEDVI (global end-diastolic volume index) showed significant predictive value.

**Discussion:**

Overall, XGBoost machine learning model based on PiCCO hemodynamic parameters provide evidence that effectively predict key clinical prognostic indicators in the patients with cardiogenic shock. These results provide important theoretical basis for further individualized clinical decision-making in cardiogenic shock patients.

## 1 Introduction

Cardiogenic shock is a critical condition characterized by cardiac dysfunction resulting in an inadequate cardiac output (1–3). It is associated with substantial morbidity and mortality (2–4). Despite significant advances in etiological treatment, pharmacological therapy, and mechanical circulatory support, the diagnosis and treatment of cardiogenic shock continue to present formidable clinical challenges.

Previous studies have mainly guided the management of cardiogenic shock through clinical manifestations and biochemical indicators, emphasizing the identification of “warm/cold” and “dry/wet” phenotypes (5, 6). However, the clinical manifestations and biochemical indicators of most patients lag behind the dynamic changes of hemodynamic parameters (6). Therefore, the dynamic hemodynamic assessment, accurate risk stratification, and timely therapeutic intervention are essential for improving clinical outcomes. Previous guidelines and expert consensus have suggested that invasive hemodynamic monitoring should be performed for patients with critical conditions or those whose symptoms do not improve significantly after initial optimized treatment (3, 7–9).

Pulse index continuous cardiac output (PiCCO) monitoring, based on transpulmonary thermodilution curves and arterial pulse contour analysis, can provide comprehensive, continuous, real-time, and accurate hemodynamic parameters (10, 11). This minimally invasive technology helps evaluate the myocardial contractility, preload, afterload, and pulmonary edema, and facilitates dynamic adjustment of treatment strategies based on monitoring results, aiming to quickly establish and maintain a stable circulatory state (10, 11). Our previous study have confirmed that compared with treatment regimens based on conventional biomarkers or scoring systems, PiCCO-guided management exerts a beneficial effect on therapeutic decision-making and improves clinical outcomes and cardiac function in patients with cardiogenic shock (12).

Compared with the classic pulmonary artery catheter, PiCCO has the following significant advantages: (1) The PiCCO system is less invasive, avoiding severe complications related to the operation. Patients with cardiogenic shock often have an indwelling central venous catheter, and the monitoring loop can be completed by only inserting an additional dedicated 4-French arterial catheter; (2) PiCCO provides continuous hemodynamic parameters, helping clinicians dynamically monitor real-time changes in the entire circulatory system and adjust treatment strategies in a timely manner; (3) Extravascular lung water index (EVLWI), a unique parameter of PiCCO, is very effective for fluid management in patients with cardiogenic shock and is closely related to the prognosis. Nonetheless, the interpretation of this multidimensional data in a time-sensitive clinical setting remains complex and highly dependent on clinician experience.

Recent advances in artificial intelligence (AI), particularly machine learning (ML), has demonstrated great promise in handling complex clinical datasets, discovering nonlinear relationships, and generating predictive models for precision medicine (13, 14). The integration of artificial intelligence into hemodynamic monitoring represents a significant advancement in the field (13–15). XGBoost is a novel explainable AI technique, which has excellent predictive capabilities in cardiovascular diseases such as myocardial infarction and related biomarkers (16–26). By integrating PiCCO-derived hemodynamic parameters with machine learning algorithms, this study aims to develop a predictive model for assessing heart failure-related clinical indicators—such as left ventricular ejection fraction (LVEF), N-terminal pro-brain natriuretic peptide (NT-proBNP) levels, and 30-day major adverse cardiovascular events (MACE) in patients with cardiogenic shock.

## 2 Methods

### 2.1 Study design and population

In this retrospective observational study, we enrolled 200 patients diagnosed with cardiogenic shock who underwent hemodynamic monitoring using the PiCCO system (Pulsion Medical Systems, Munich, Germany). All participants were admitted to the Cardiac Care Unit (CCU) of Shanghai East Hospital between January 2024 and May 2025.

The inclusion criteria were as follows: (1) age ≥ 18 years old and (2) patients diagnosed with cardiogenic shock. The diagnosis of cardiogenic shock was based on the following criteria according to the guidelines: (1) objective evidence of cardiac dysfunction; (2) systolic blood pressure < 90 mmHg or mean arterial pressure < 60 mmHg for at least 30 min, or requiring vasopressor agents or intra-aortic balloon pump (IABP) to maintain blood pressure above these thresholds; (3) cardiac index (CI) < 2.2 L/(min·m^2^); and (4) clinical signs of tissue hypoperfusion (cold, clammy skin, altered mental status, oliguria or peripheral vasoconstriction, etc.).

The exclusion criteria included the following: (1) severe peripheral vascular disease; (2) significant valve heart disease; (3) treatment with mechanical circulatory support (MCS), including extracorporeal membrane oxygenation (ECMO), Impella, TandemHeart, or left ventricular assist device (LVAD); (4) complicated with severe infection or acute respiratory distress syndrome; and (5) pregnancy or lactation.

### 2.2 Interventions

The hemodynamic monitoring was performed by experienced intensivists following standard protocols. In addition to inserting a central venous catheter via the internal jugular or femoral vein, a PiCCO arterial catheter was placed in the femoral artery. The pressure transducer and temperature probe were connected to the machine module according to the operation manual. For cardiac output measurement, the zero point was first calibrated, and then 15–20 mL of 0–4 °C saline solution was injected via the venous catheter 3–5 times, with the average values recorded and calculated. Calibration was performed at least every 8 h to ensure the accuracy of the data. The hemodynamic parameters were continuously recorded until the patient either resolved from cardiogenic shock or death occurred (27).

### 2.3 Data collection

Clinical data were extracted from the electronic medical record system, including demographic characteristics, laboratory values (e.g., NT-proBNP), echocardiographic findings (e.g., LVEF), and 30-day MACE, defined as the composite of all-cause mortality, cardiac mortality, and heart failure readmission.

Hemodynamic parameters obtained via the PiCCO system included: CI (cardiac index), CPI (cardiac power index), CFI (cardiac function index), GEF (global ejection fraction), dPmax (index of the left ventricular contractility), GEDVI (global end-diastolic volume index), CVP (central venous pressure), PPV (pulse pressure variation), SVV (stroke volume variation), EVLWI (extravascular lung water index), PVPI (pulmonary vascular permeability index), SVRI (systemic vascular resistance index) (28).

The selection of PiCCO hemodynamic parameters was based on their established clinical relevance in reflecting cardiac function, preload, afterload, and pulmonary edema in cardiogenic shock patients. For instance, CI, CPI, CFI, GEF and dPmax are direct indicators of cardiac function; GEDVI and CVP reflect volume status; PPV and SVV are the parameters for volume responsiveness; SVRI represents afterload; EVLWI and PVPI indicate pulmonary edema risk. These parameters are routinely used in critical care settings to guide fluid management, inotropic support, and vasopressor therapy. Their inclusion in the model aligns with current hemodynamic monitoring guidelines and clinical practices, ensuring that the predictive features are physiologically interpretable and actionable.

All PiCCO measurements were obtained within the first 24 h of a patient’s CCU admission to reflect the early hemodynamic characteristics of cardiogenic shock.

Outcome data were collected by trained medical professionals either during hospitalization or through outpatient follow-up. All data were retrieved from electronic medical records and anonymized to ensure patient confidentiality. Two independent cardiologists determined MACE based on medical records and follow-up; any disagreement was arbitrated by a third senior physician. Readmissions were limited to unplanned hospitalizations related to heart failure decompensation. Mortality information was obtained from in-hospital records or authoritative follow-up sources, clarifying the principles of “loss to follow-up.”

### 2.4 AI model establishment and training process

For the AI model’s establishment and training process, the XGBoost machine learning model was selected. XGBoost machine learning model is an optimized distributed gradient boosting library that provides better predictive power by transforming a set of weak learners into strong learners. The algorithm is powerful due to several innovations such as approximate greedy search, parallel learning, and hyperparameters (26, 29). This study selected the XGBoost model to build a prediction AI model, which was applied to: (1) Multi-class prediction of LVEF levels. LVEF were stratified into five classes: ≤20, 21–30%, 31–40%, 41–50%, ≥50%; (2) Multi-class prediction of NT-proBNP stratification. NT-proBNP levels were stratified into four classes: <10,000 ng/L, 10,001–20,000 ng/L, 20,001–30,000 ng/L, and >30,000 ng/L; (3) Binary classification prediction of 30-day MACE events occurrence. The XGBoost model has advantages such as efficiency, scalability, and strong regularization capabilities, and is suitable for processing high-dimensional non-linear structured data sets. To optimize model performance and minimize overfitting, hyperparameter tuning was conducted using a grid search strategy, exploring learning rate, maximum tree depth, number of estimators, subsample ratio, column sampling, and L1/L2 regularization terms. The dataset was randomly divided into training (50%) and testing (50%) cohorts, with 5-fold cross-validation performed within the training set. Overfitting was mitigated by incorporating early stopping, shrinkage through learning rate adjustment, and regularization penalties, thereby ensuring robust and generalizable model performance.

### 2.5 AI model interpretability analysis

To enhance the clinical interpretability of the model, the SHapley Additive exPlanations (SHAP) algorithm was employed to conduct a feature importance analysis of the model output. This method is based on the Shapley value calculation principle in game theory and can reveal the contribution direction and magnitude of each feature to the prediction results from both a global and local perspective (30, 31).

By drawing SHAP beeswarm diagrams, feature influence ranking diagrams (bar plots), and global feature influence heatmaps (summary plots), the roles of key variables such as CI, dPmax, and GEDVI in the model prediction can be clearly demonstrated. This provides a theoretical basis for further optimizing feature selection and simplifying the model in clinical practice.

### 2.6 AI model evaluation and comparison

The LVEF and NT-proBNP model uses the multi-class ROC curve and its AUC (Area Under Curve) value as the main evaluation indicators. The MACE model uses ROC-AUC, accuracy, sensitivity, and specificity for evaluation. Calibration analysis assesses the consistency between the predicted probabilities output by the model and the actual events by drawing a calibration curve. Comparative analysis compares the performance of the XGBoost model with that of Logistic regression to verify its advantages in high-dimensional data scenarios (24, 32–34).

## 3 Results

### 3.1 Analysis of hemodynamic monitoring by PiCCO and prediction of LVEF based on XGBoost model

In this study, the XGBoost machine learning model was used to analyze the relationship between hemodynamic indicators by PiCCO and LVEF in cardiogenic shock patients. By using various visualization tools, we comprehensively explored the prediction effect and feature contribution of the model to deeply understand the influence of each feature on the prediction of LVEF.

**Figure 1:**
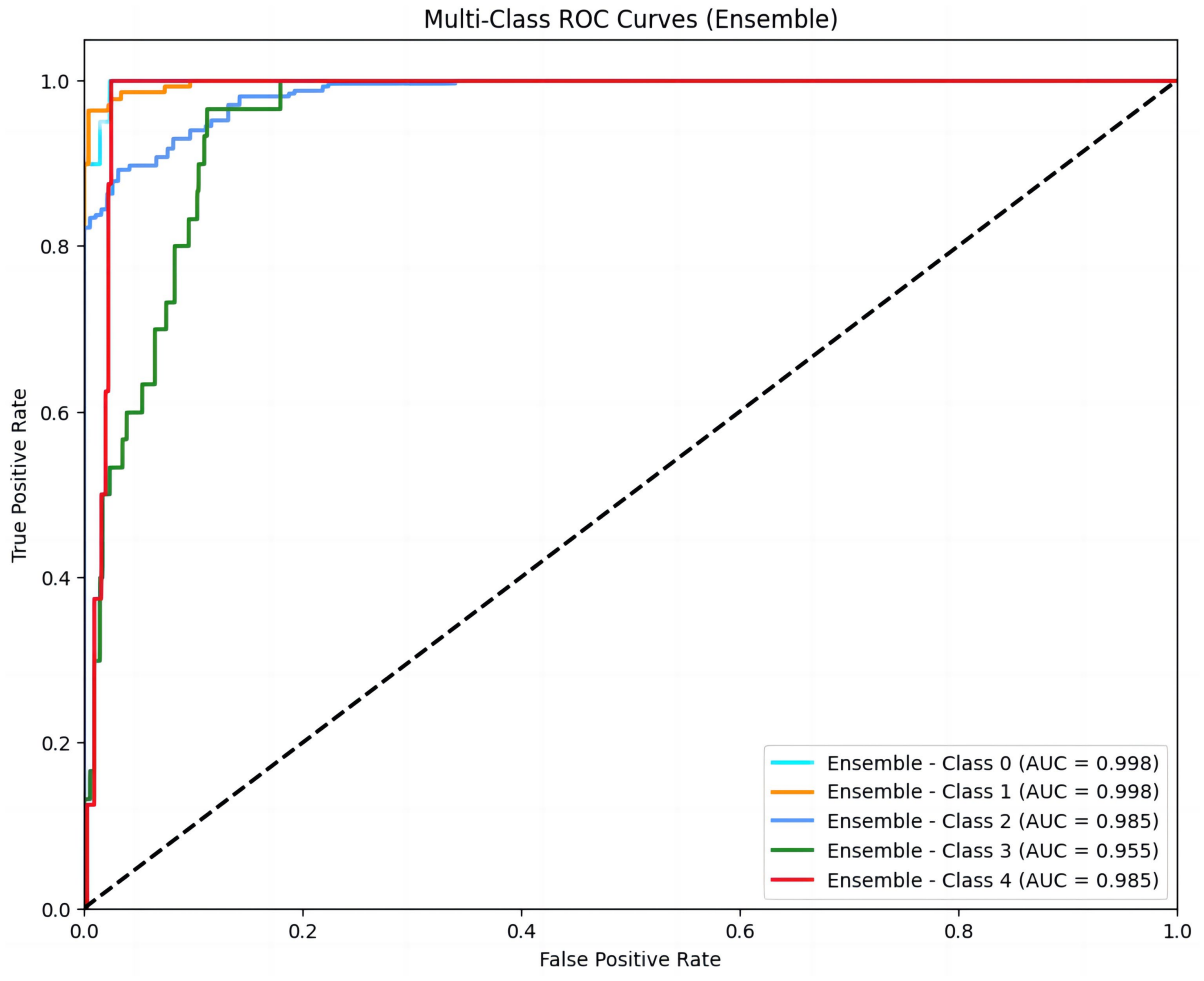
Multi-class ROC curves for LVEF classification prediction. The figure displays ROC curves for five LVEF classes (Class 0 to Class 4), representing the model’s performance across different thresholds. The area under the curve (AUC) values (0.998, 0.998, 0.985, 0.955, 0.985) indicate excellent discriminatory ability for each class. Higher AUC values indicate stronger discriminatory ability of the model. LVEF were stratified into five classes: ≤20, 21–30%, 31–40%, 41–50%, ≥50%.

To explore the strong classification ability and good generalization performance of the XGBoost model in the LVEF prediction task, we first constructed multi-class ROC curves. The ROC curve shows the model’s performance at different classification thresholds, with the x-axis representing the false positive rate (FPR) and the y-axis representing the true positive rate (TPR). This result demonstrated the performance of ROC curves for different classes (Class 0, Class 1, Class 2, Class 3, Class 4). The area under the curve (AUC) values of each curve were 0.998, 0.998, 0.985, 0.955, and 0.985, respectively, indicating the excellent performance of the model in LVEF classification prediction. Among them, the AUC values of class 0 and class 1 were close to 1, indicating that the model had a strong discrimination ability for these two classes and predicted LVEF levels with a relatively high degree of accuracy (Figure 1).

**Figure 2:**
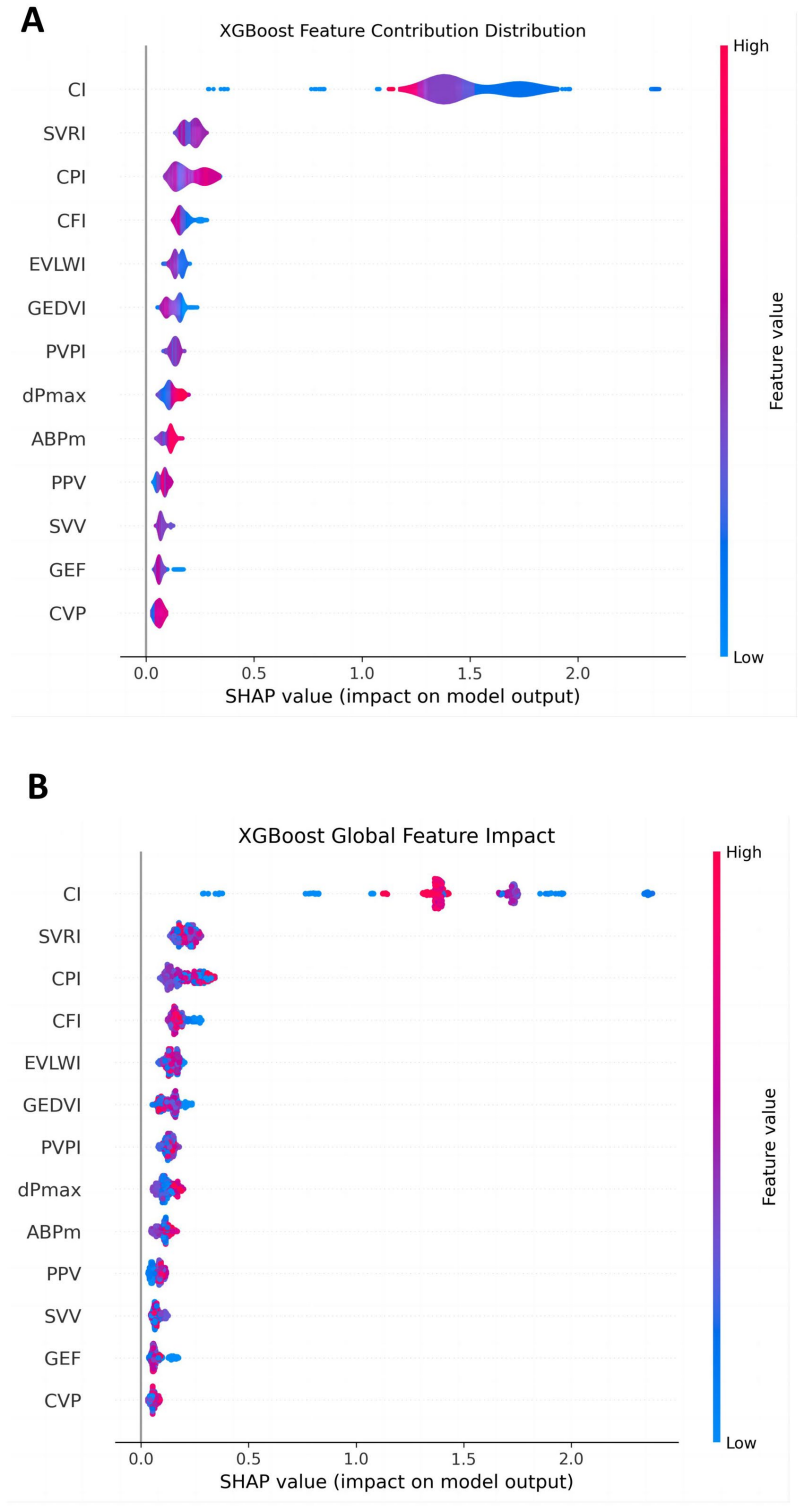
Contribution of each feature in the LVEF prediction model. **(A)** The overall impact of each feature; **(B)** the detailed contribution of each feature for every individual patient. The *X*-axis shows the SHAP value, indicating the magnitude and direction (positive or negative) of each feature’s impact on LVEF prediction. Color changes reflect the magnitude of the feature value (blue: low, red: high). The larger the absolute SHAP value, the greater the feature’s impact on the model’s prediction accuracy. For example, features such as CI, SVRI, and CPI show high contributions, while SVV, GEF and CVP have minimal impact.

In order to explore the contribution of each PiCCO hemodynamic parameters to the output of the XGBoost model, we used SHAP for further evaluation. The SHAP value is typically used to indicate the presumed influence degree of each feature on the model output. The SHAP value of each feature is displayed horizontally, with colors ranging from blue (low value) to red (high value), indicating the size of the feature value. Generally, the larger SHAP value represents the greater influence of the feature on the prediction result. As can be seen from the figure, features such as CI, SVRI, CPIand CFI had a significant positive effect on the model output. In particular, when these feature values were high, the accuracy of LVEF prediction was significantly improved. On the other hand, features such as SVV, GEF and CVP had relatively low impact on the model output, indicating that their contribution to the LVEF prediction results was relatively small. This showed that the choice of features and their value ranges directly affected the prediction accuracy of the model (Figure 2A).

To further reveal the global contribution of different features to LVEF prediction, we constructed an XGBoost global feature influence figure (Figure 2B). This figure showed the distribution of SHAP values for each PiCCO hemodynamic parameter. It can be seen that features such as CI, SVRI, CPI and CFI had a more significant impact across the entire dataset, especially when their values were high, contributing most significantly to LVEF prediction. In contrast, features such as CVP, GEF and SVV had a smaller impact, with a more dispersed distribution of SHAP values, indicating that these features had a less pronounced impact on the overall model. This provides guidance for further optimizing model feature selection and identifying which features seem to constitute the key factors in predicting LVEF.

These results offer a deeper understanding of the PiCCO parameters play an important role in predicting LVEF among patients with cardiogenic shock. Multi-class ROC curves demonstrate the model predictive power for different LVEF categories. Contribution distribution plots and global influence diagrams also reveal the crucial role of different PiCCO metrics in model prediction. These findings provide important theoretical support for future model optimization and personalized prediction.

### 3.2 Prediction analysis of PiCCO hemodynamic parameters and NT-proBNP levels based on XGBoost model

**Figure 3:**
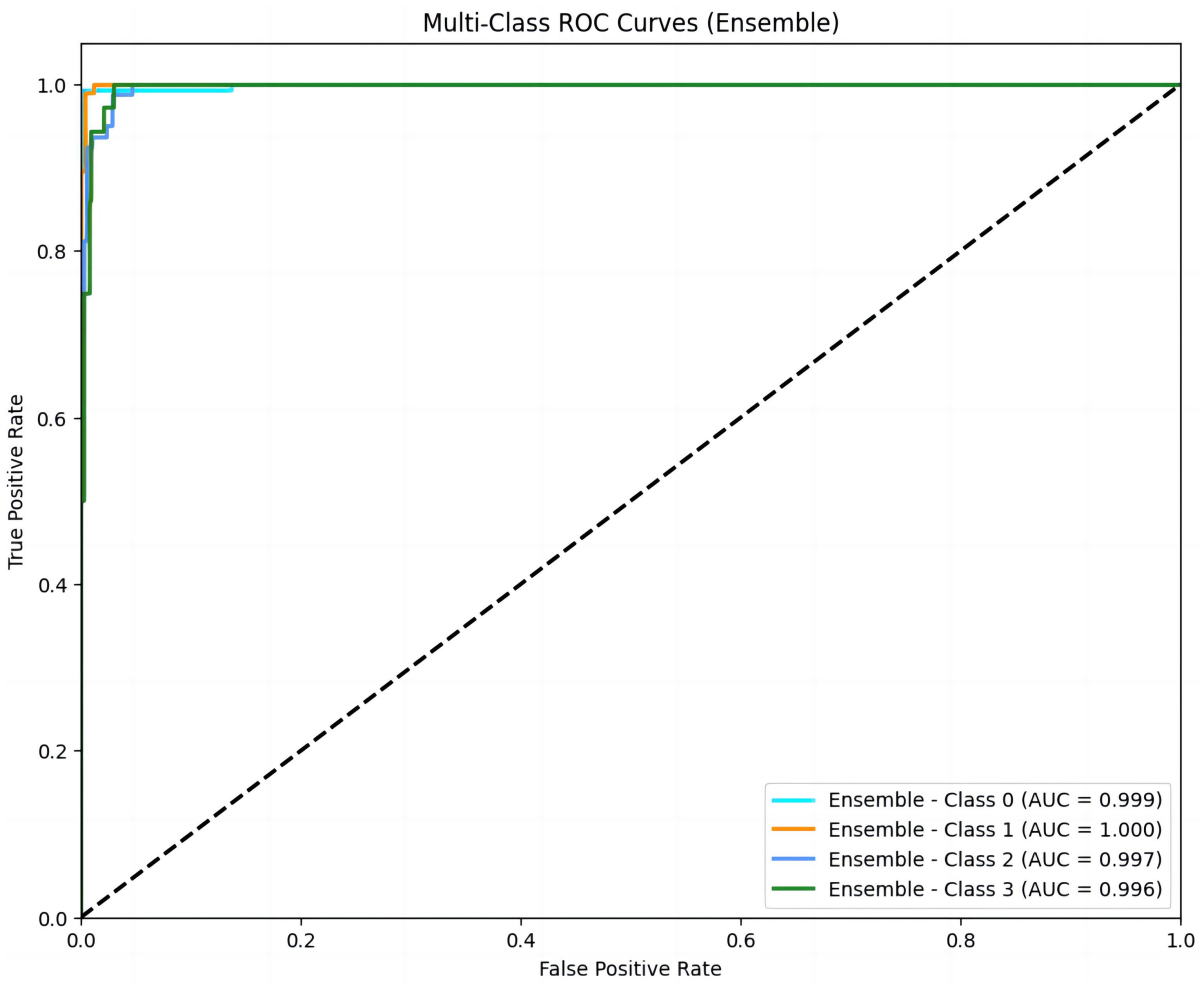
Multi-class ROC curves for NT-proBNP prediction. ROC curves for four NT-proBNP classes (Class 0 to Class 3) demonstrate the model’s high predictive accuracy, with AUC values of 0.999, 1.000, 0.997, and 0.996. An AUC value close to 1 indicates a high predictive accuracy of the model. NT-proBNP levels were stratified into four classes: <10,000 ng/L, 10,001–20,000 ng/L, 20,001–30,000 ng/L, and >30,000 ng/L.

We also constructed a multi-class ROC curve to analyze the accuracy of the relationship between PiCCO parameters and NT-proBNP levels. The AUC values for class 0, class 1, class 2, and class 3 were 0.999, 1.000, 0.997, and 0.996, respectively, demonstrating that the model performed well in predicting NT-proBNP. In particular, the AUC for class 1 was 1.000, indicating high predictive accuracy for this category. Additionally, the AUC values for other categories were also very close to 1. Therefore, the XGBoost model could distinguish different NT-proBNP levels and possessed a high classification ability (Figure 3).

**Figure 4:**
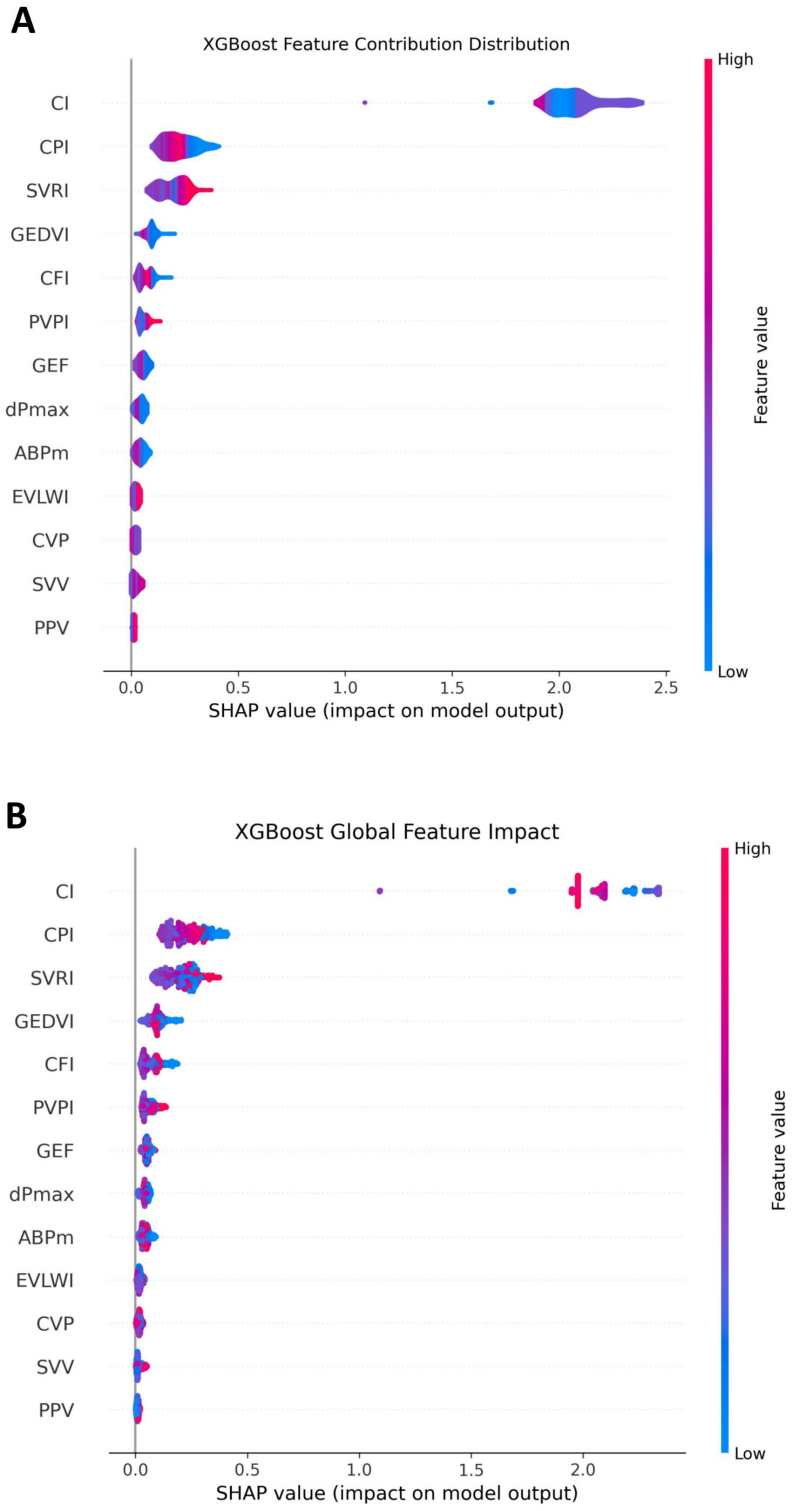
Contribution of each feature in the NT-proBNP prediction model. **(A)** The overall impact of each feature; **(B)** the detailed contribution of each feature for every individual patient. SHAP values represent the change in predicted NT-proBNP level relative to the baseline. CI, CPI, and SVRI show strong effects when values are high, whereas CVP, SVV, and PPV have limited influence.

The SHAP values figure demonstrated that features such as CI, CPI, and SVRI played a significant role in influencing the model output, especially when their values were high, which significantly increased the model output. In contrast, features like CVP, SVV, and PPV had a relatively small contribution to the model, showing a relatively concentrated influence. This indicated that CI, CPI, and SVRI features had important positive or negative effects on NT-proBNP prediction, while the influence of other features was relatively weak (Figure 4A).

From a global perspective, we observed that some features such as CI, CPI, and SVRI had a significant impact throughout the dataset, and their SHAP values were mostly concentrated in a high range, indicating their significant contribution to predicting NT-proBNP. In contrast, features like CVP, SVV, and PPV had a relatively small influence on a global scale and having an insignificant impact. This provides a strong basis for identifying the most predictive features in the model (Figure 4B).

### 3.3 Prediction analysis of PiCCO hemodynamic parameters and 30-day MACE based on XGBoost model

This research also used the XGBoost machine learning model to clarify the relationship between invasive PiCCO hemodynamic parameters and 30-day MACE, including all-cause mortality, cardiac mortality, and heart failure readmission. As an important clinical event, the prediction of MACE is of great significance for the early intervention of patients.

**Figure 5:**
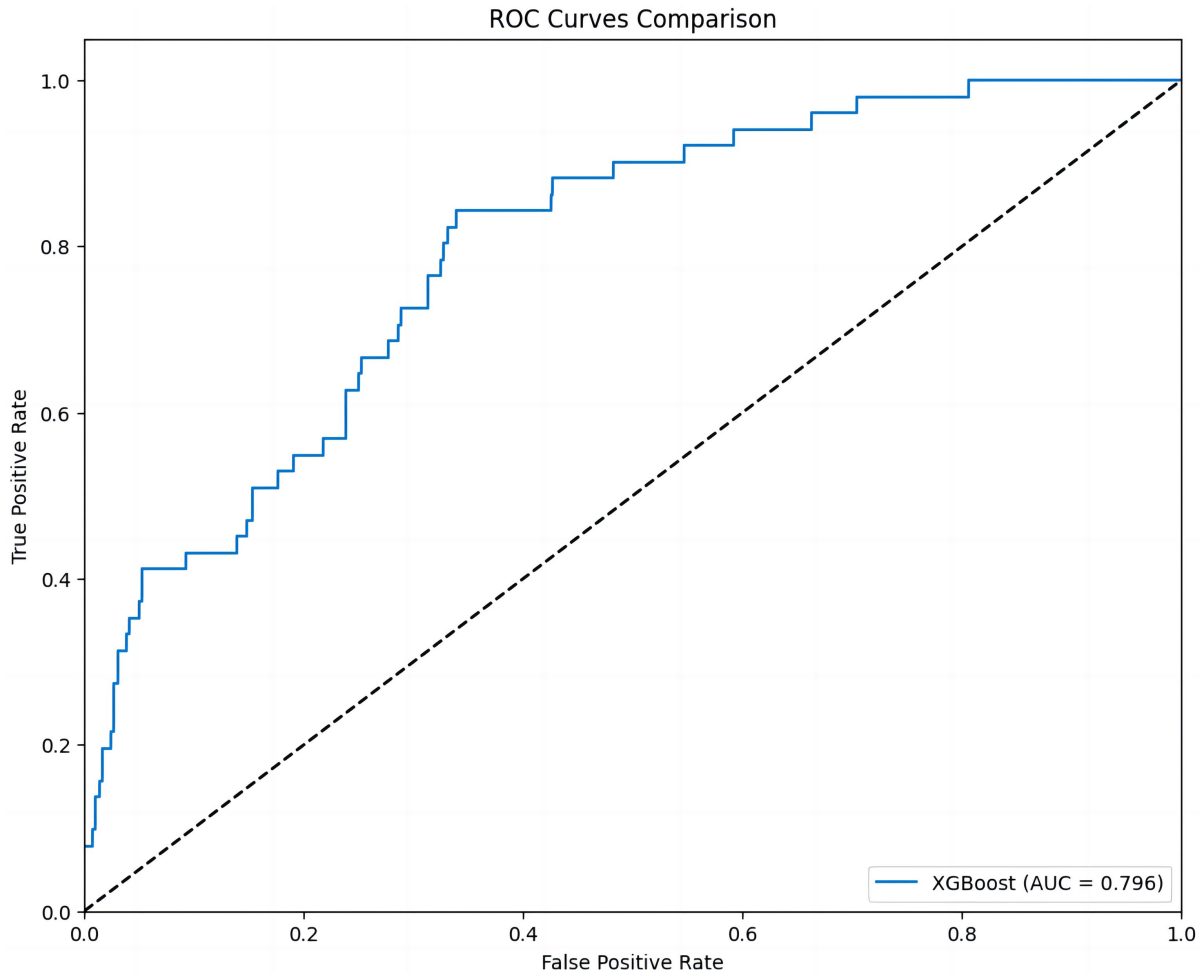
ROC curve of the 30-day MACE prediction model. This figure shows the ROC curve of the XGBoost model. The AUC value is 0.796, indicating that the model has strong discriminatory power in predicting different MACE outcomes.

The ROC curve provided an intuitive assessment of the prediction model. The area under the curve (AUC) value of the curve was 0.796, indicating good but not optimal discrimination between patients with and without adverse events (Figure 5).

**Figure 6:**
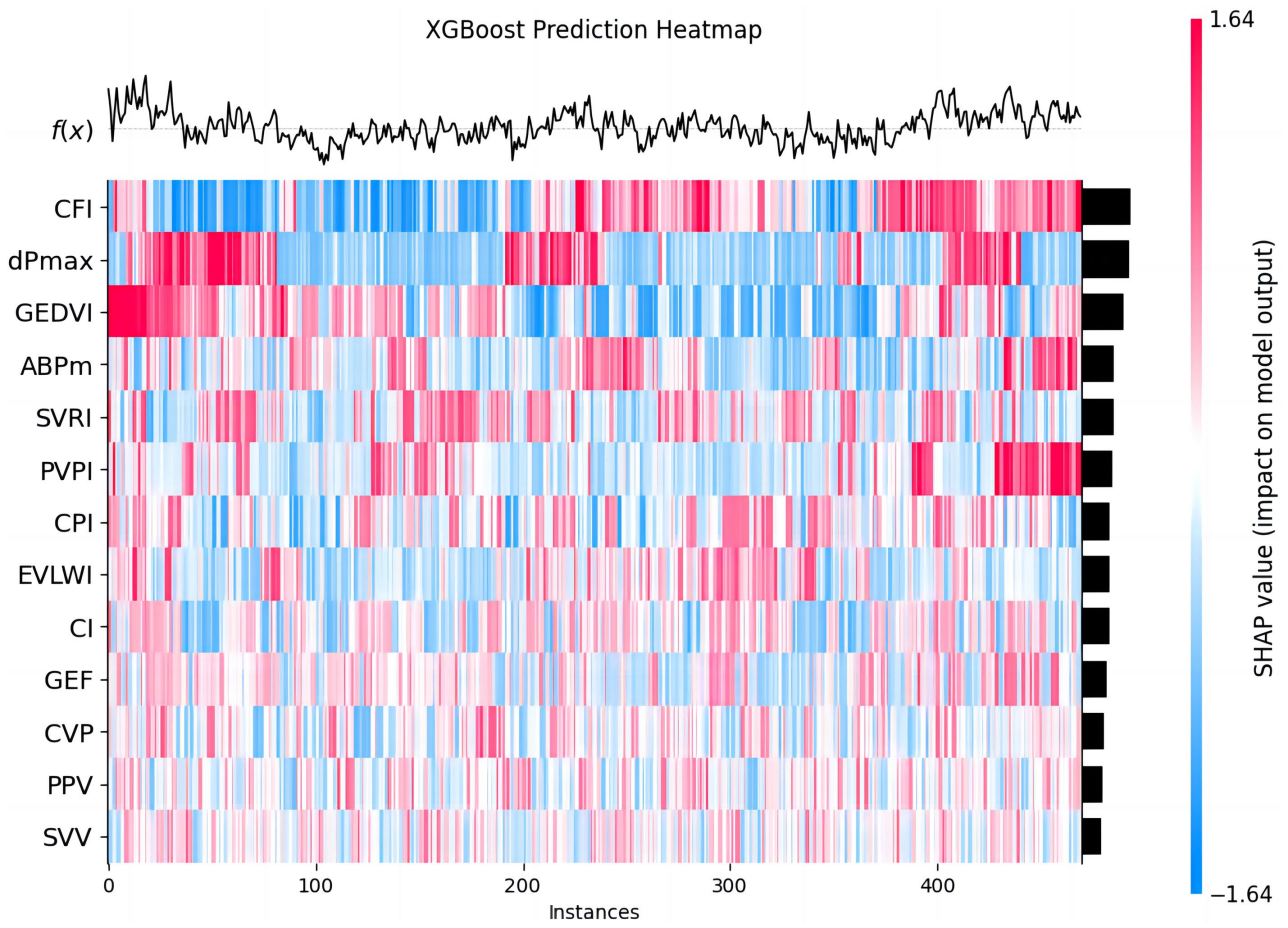
The predictive effect of each feature in 30-day MACE prediction. This chart shows the predictive effect of each feature in the XGBoost model on different data instances. Each row represents a feature, each column a patient instance. The colors from blue to red represent the range of SHAP values (blue: negative impact, red: positive impact). The heatmap visualizes how each feature variably influences MACE prediction across the cohort.

The heatmap was constructed to observe how the influence of different features, such as CFI, dPmax, and GEDVI, varied across the dataset, and helped us understand how each feature impacts the prediction for each instance. The color of heatmap gradually changes from blue to red, indicating the change in SHAP values, further revealing the predictive power of different features at different data points (Figure 6).

**Figure 7:**
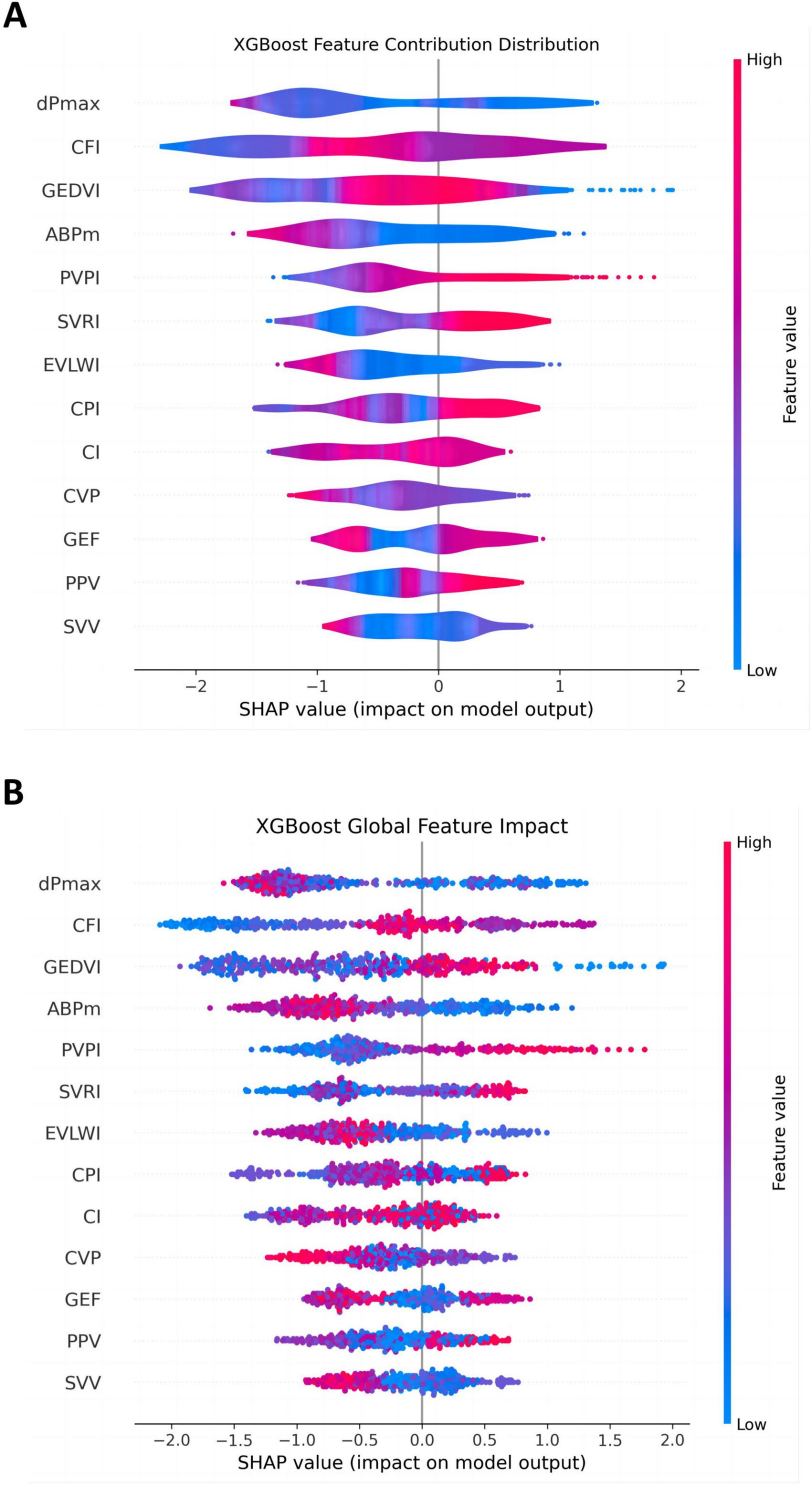
Distribution of feature contributions in the 30-day MACE prediction model. This figure shows the distribution of SHAP values for each feature in the XGBoost model, reflecting the contribution of these features to the model output. **(A)** The overall impact of each feature; **(B)** the detailed contribution of each feature for every individual patient. Features like dPmax, CFI, and GEDVI have wide distributions, indicating their variable but significant impact. SVV and PPV show narrower distributions, suggesting minor effects.

To further analyze the model, the SHAP values for each feature were used to illustrate the degree to which different feature values influence the model output. Features with larger SHAP values, such as dPmax, CFI, and GEDVI, had a wider distribution, indicating that these features contributed more significantly to the prediction results under different circumstances. In contrast, other features, such as GEF, PPV, and SVV, had a smaller impact on the model output and their SHAP values were more concentrated, indicating that their contribution to the prediction results is weaker (Figure 7A).

The global feature influence plot showed the impact of each PiCCO parameter on MACE, helping us understand the importance of each parameter across the entire dataset (Figure 7B). In this figure, features such as dPmax, CFI, and GEDVI played a more significant role in model prediction. In contrast, some features, such as GEF, PPV and SVV, had a relatively small impact on the model. This global perspective allows us to further understand feature importance and provide guidance for future model optimization and feature selection.

**Figure 8:**
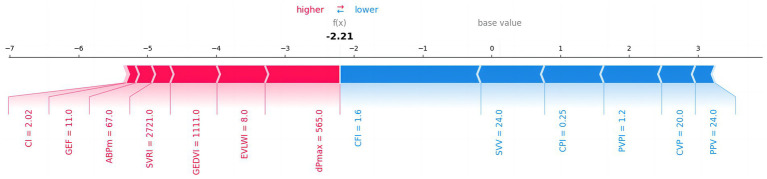
The impact of each feature on 30-day MACE prediction results. The figure shows the influence of different features on the model’s prediction results. Red indicates a positive impact on the prediction, while blue indicates a negative impact.

The SHAP value summary figure showed the contribution of each feature to the model prediction results. SHAP values help us understand how each PiCCO parameters affects the model output. Red in the chart indicates a positive impact of the feature value on the prediction result, while blue indicates a negative impact. SHAP summary figure indicated that dpmax and GEDVI exerted positive effects on prediction scores, whereas SVV and PPV were associated with negative contributions (Figure 8).

Therefore, through these figures, we have gained a deep understanding of the influence of each PiCCO hemodynamic parameters on the prediction of MACE results in the XGBoost model. SHAP value analysis, ROC curves, and feature contribution distribution figure help us comprehensively evaluate the performance of the XGBoost-based PiCCO hemodynamic parameters and MACE prediction model, as well as its reliability in predicting MACE. These results provide important insights for further model optimization and improved prediction accuracy in cardiogenic shock patients.

## 4 Discussion

LVEF, NT-proBNP can facilitate prompt recognition, diagnosis, and management of shock, ultimately improving patient outcomes (35–38). However, the biochemical indicators lag behind the dynamic changes of hemodynamic parameters (6). Hemodynamic status is a critical determinant of both risk stratification and clinical prognosis in cardiogenic shock (39, 40). ML-based phenotyping is playing a growing role in risk stratification and therapeutic decision-making for cardiogenic shock (41–44). This research lies at the forefront of critical care cardiology and embodies the convergence of state-of-the-art hemodynamic monitoring with cutting-edge computational approaches. By leveraging AI-driven analytics, our model has the potential to uncover clinically actionable insights, provide real-time risk prediction, and guide individualized treatment strategies. This work offers a novel direction for the diagnosis and management of cardiogenic shock, enhances the precision and personalization of care for critically ill cardiovascular patients, and ultimately aims to improve prognosis, reduce mortality, and lower healthcare costs. The proposed model not only holds significant clinical value but also carries important societal and economic implications in the era of intelligent medicine.

In recent years, XGBoost-based machine learning prediction models have been widely used in the medical field, demonstrating remarkable predictive performance in cardiovascular disease, sepsis, and kidney injury management (19–26, 45–47).

This study utilized the XGBoost algorithm to systematically evaluate the predictive value of PiCCO-derived hemodynamic parameters for LVEF, NT-proBNP, and MACE in patients with cardiogenic shock. Our results showed that the XGBoost model achieved extremely high classification accuracy in multiple prediction tasks. Especially, The AUC values of the ROC curves in the predictions of LVEF and NT-proBNP are close to 1, indicating a strong discriminative capacity even in a complex and unstable hemodynamic setting (48).

Through SHAP value analysis, we further revealed the contribution degree of each hemodynamic parameter to the model’s prediction results. CI, SVRI, and CPI had significant influences on the prediction of LVEF and NT-proBNP, indicating that these parameters should be given high attention in clinical practice (49). However, parameters such as PPV, SVV and CVP had relatively weak influences on the prediction. CI reflects global cardiac output adjusted for body surface area, directly mirroring the extent of myocardial contractile impairment. In cardiogenic shock, reduced CI indicates severe cardiac dysfunction and inadequate tissue perfusion. After identifying the specific causes of the reduced cardiac index—considering myocardial contractility, preload, afterload, and pulmonary edema status—we can apply a hemodynamic and volume management decision model to guide inotrope titration, vasoactive therapy, and fluid management (12). SVRI reflects afterload and vascular tone, both of which are profoundly influenced by neurohormonal activation and vasopressor use during cardiogenic shock. CPI is a comprehensive indicator representing the pump and work capacity of the heart, and it is a core parameter for measuring cardiac function. From a pathophysiological perspective, the key predictive features of these parameters reveal the importance of improving myocardial contractility, preload, and afterload in the treatment of cardiogenic shock. Deteriorated CPI is a strong prognosticator of mortality in cardiogenic shock and helps stratify high-risk patients requiring more aggressive mechanical circulatory support.

For 30-day MACE prediction, although the AUC (0.796) was lower than that for LVEF and NT-proBNP, it was still at a good prediction level, and the key influencing features identified by the model (such as dPmax, CFI, GEDVI) were closely related to cardiac function and had high clinical relevance (50). This finding aligns with the understanding that persistent contractile dysfunction (low dPmax, reduced CFI) and inappropriate preload (abnormal GEDVI) are critical determinants of adverse outcomes in CS survivors. dPmax and CFI are sensitive indicators of cardiac contractility and its response to inotropic drugs. They can provide real-time insight into myocardial inotropy and guide optimization of inotropic therapy. GEDVI serves as a preload marker and help differentiate hypovolemia from fluid overload, informing tailored volume management strategies. These parameters likely capture residual myocardial dysfunction and suboptimal hemodynamic profiles that predispose patients to recurrent heart failure or death. This model serves as a powerful auxiliary tool to quickly and accurately identify high-risk patients. The key indicators require more attention to cardiogenic shock patients with low dPmax, low CFI, and high GEDVI. Our results indicate that the multi-dimensional parameters provided by the PiCCO system can not only reflect the current hemodynamic state but also predict the risk of future adverse events to a certain extent.

The results of this study support the integration of machine learning methods into clinical hemodynamic monitoring, helping doctors extract valuable information from high-dimensional and complex parameters and promoting the formulation of individualized treatment decisions for cardiogenic shock patients. This approach not only allows for real-time, individualized risk prediction but also has the potential to guide targeted interventions—such as preload optimization, afterload modulation, or tailored inotropic support—before irreversible organ injury occurs.

## 5 Conclusion

This study constructed and validated the application of an XGBoost machine learning model based on PiCCO monitoring parameters for predicting LVEF, NT-proBNP levels, and MACE in patients with cardiogenic shock. Results showed that the model demonstrated high discriminative power for both LVEF and NT-proBNP grading, and also demonstrated good discriminative power for MACE prediction. SHAP interpretability analysis revealed that key hemodynamic parameters, including CI, CPI, SVRI, dPmax, CFI and GEDVI contributed significantly to the predictions, suggesting their importance in clinical risk assessment.

The results demonstrate that combining machine learning methods with dynamic monitoring data can effectively exploit potential nonlinear relationships, thereby improving the accuracy of early warning and personalized decision-making. This not only helps optimize diagnostic and treatment strategies for patients with cardiogenic shock but also provides a methodological basis for the development of future intelligent intensive care systems.

### 5.1 Limitations and perspective

Firstly, a single-center retrospective study design with a relatively modest sample size may impair the generalizability and increase the risk of overfitting. Secondly, external validation on independent multicenter datasets is warranted to confirm robustness and reproducibility. Thirdly, although PiCCO monitoring provides real-time and accurate hemodynamic parameters, its limitations restrict its application in all patients with cardiogenic shock, including invasiveness, high cost, operator dependency, and uncapable in patients undergoing advanced MCS. Last but not least, the AUC value of the MACE prediction model (0.796) is lower than LVEF and NT-proBNP, while within the acceptable range (>0.7). This may be due to the fact that MACE, as a composite endpoint, is influenced by multiple non-hemodynamic factors. Previous studies have also confirmed that machine learning makes limited contributions to clinical endpoints including mortality, but the models improved risk stratification for high-risk individuals (26).

Future research can expand model training on prospective, multi-center, multi-population data and attempt to integrate more advanced algorithms such as graph neural networks to further improve prediction accuracy and interpretability. Additionally, it is important to develop hybrid predictive models that combine noninvasive biomarkers (e.g., troponin, lactic acid, and echocardiographic parameters) with PiCCO-derived hemodynamic indices. Moreover, embedding these models into a real-time bedside decision-support system could facilitate dynamic risk stratification and personalized management of cardiogenic shock patients.

## Data Availability

The raw data supporting the conclusions of this article will be made available by the authors, without undue reservation.
